# Common variants in adiponectin gene are associated with coronary artery disease and angiographical severity of coronary atherosclerosis in type 2 diabetes

**DOI:** 10.1186/1475-2840-12-67

**Published:** 2013-04-17

**Authors:** Guoxin Tong, Ningfu Wang, Jianhang Leng, Xiaoshan Tong, Yun Shen, Jianmin Yang, Xianhua Ye, Liang Zhou, Yujie Zhou

**Affiliations:** 1 Department of Cardiology, Beijing Anzhen Hospital, Capital Medical University, Beijing, China; 2 Department of Cardiology, Hangzhou Hospital (Hangzhou No.1 Municipal Hospital), Nanjing Medical University, Hangzhou, China; 3 Department of Clinical Laboratory Medicine, Hangzhou Hospital (Hangzhou No.1 Municipal Hospital), Nanjing Medical University, Hangzhou, China

**Keywords:** Adiponectin, Genetics, Coronary artery disease, Angiography, Gensini score, Sullivan Extent score, Single nucleotide polymorphism

## Abstract

**Background:**

Adiponectin, an adipokine facilitating insulin action, has antiatherogenic effects. This study investigated whether common single nucleotide polymorphisms (SNPs) in the adiponectin gene influenced plasma adiponectin level and whether they were associated with the risk of coronary artery disease (CAD) and its angiographical severity in type 2 diabetes in Chinese population.

**Methods:**

11 tagging SNPs were genotyped in 1110 subjects with or without CAD in type 2 diabetes. Variants of adiponectin gene were determined by Taqman polymerase chain reaction method. The plasma adiponectin concentrations were measured by sandwich enzyme-linked immunosorbent assay. The severity and extent of coronary atherosclerosis were assessed using the angiographic Gensini score and Sullivan Extent score.

**Results:**

Among the 11 SNPs, the minor G allele of SNP rs266729 was significantly associated with higher odds of CAD (odds ratio (95% CI) = 1.49 (1.10 - 2.16), P = 0.022) after adjusting for covariates. In stepwise multivariate logistic regression, SNP rs266729 was a significant independent factor of CAD. Multivariate linear regression analysis revealed that rs266729 (β = −0.101, P < 0.0001), rs182052 (β = −0.044, P = 0.0035), and rs1501299 (β = 0.073, P < 0.0001) were significantly associated with adiponectin level, and also indicated that the minor G allele of SNP rs266729 had higher Gensini score (β = 0.139, P < 0.001) and Sullivan Extent score (β = 0.107, P < 0.001). Haplotypes analysis revealed different haplotype distributions in case and control subjects (P = 0.0003), with two common haplotypes GGG and GAG of the rs266729, rs182052, and rs1501299 being associated in heterozygotes with a greater than threefold increase in cardiovascular risk (odds ratio (95% CI)=3.39 (1.83 - 6.30), P = 0.0001).

**Conclusions:**

In our population, genetic variants in the adiponectin gene influence plasma adiponectin levels, and one of them is a strong determinant of CAD susceptibility and its angiographical severity in type 2 diabetes. This study has provided further evidence for a role of adiponectin in the development of CAD.

## Background

Adiponectin is an adipose tissue-derived adipocytokine protein, which has been identified in the human adipose tissue complementary DNA library [1]. Concentration of adiponectin decreases in patients with the phenotypes of the metabolic syndrome [2], including obesity [3], type-2 diabetes [4], insulin resistance [5], hypertension [6]. Lower plasma concentrations of adiponectin are also associated with coronary artery disease (CAD) [7,8].

In humans, adiponectin is encoded by portions of exons 2 and 3 among the three exons of the adiponectin, C1Q and collagen domain-containing (ADIPOQ) gene located on chromosome 3q27 [9]. The role of common polymorphisms of the adiponectin gene in cardiovascular disease has been investigated previously in several populations, such as Caucasian and Korean population [10,11]. However, inconsistent findings on the association of several genetic variants of ADIPOQ with adiponectin level [12-15] and cardiovascular disease have been reported [16,17], which could be due to a difference in ethnic populations, single nucleotide polymorphism (SNP) selection, and study power. Therefore, data regarding the relationship between adiponectin gene polymorphisms and CAD need to be further investigated because of the conflicting reported results. To our best knowledge, there is no systematic analysis of SNPs in ADIPOQ gene with regard to both adiponectin level and CAD in Chinese population.

In this study, we investigated the association of common genetic variants in the ADIPOQ gene with adiponectin level and CAD in type 2 diabetes, a condition of accelerated atherogenesis in which the presence of defects in contributing genetic factors may be especially evident, and we also investigated its association with angiographical severity of coronary atherosclerosis.

## Materials and methods

### Patients and controls

Consecutive 560 stable CAD patients and 550 control subjects with type 2 diabetes were recruited from the inpatients or outpatients who have undergone coronary arteriography for suspected or known coronary atherosclerosis at our hospital. The criteria for CAD were a ≥50% organic stenosis of at least one segment of a major coronary artery or their main branches confirmed by coronary angiography. Control subjects were the patients, aged ≥55 years, who had diabetes for ≥5 years, with coronary stenosis (at angiography) <50%. Exclusion criteria were active inflammatory conditions, autoimmune disease, malignancies, use of immunosuppressive drugs and known hematological disorders. Patients with ST elevation myocardial infarction, unstable angina or NSTEMI were also excluded. Written informed consent was obtained from all enrolled participants and this study was approved by the local Ethics Committee.

### Coronary angiography and image interpretation

Coronary angiography was carried out according to the Judkins technique. Coronary artery stenoses were imaged in the centre of the field from multiple projections. An overlap of side branches and foreshortening of relevant coronary arteries was avoided as far as possible. Coronary angiograms were scored according to Gensini score and Sullivan Extent score.

In Gensini’s scoring system [18], angiographic stenosis in the range of 0%–25% was scored as 1 point, stenosis in the range of 25%–50% was scored as 2 points, 50%–75% was scored as 4 points, 75%–90% was scored as 8 points, 90%–99% was scored as 16 points, and total occlusion was scored as 32 points. Each stenosed segment was then weighted from 0.5 to 5, depending on the functional significance of the area supplied by that segment. These scores were multiplied by the coefficient defined for each coronary artery and segment, and the results were then summed.

The Sullivan Extent score quantifies the percentage of the coronary intimal surface area affected by atheroma, as identified by lumen irregularity, was multiplied by a factor for each vessel: left main, 5; left anterior descending, 20; main diagonal branch, 10; first septal perforator, 5; left circumflex, obtuse marginal, and posterolateral vessels, 10; right coronary, 20; and main posterior descending branch, 10. When the major lateral wall branch was a large obtuse marginal or intermediate vessel, the factor used was 20, with factor of 10 for the left circumflex. Occluded vessels which were filled with contrast medium by collateral flow were evaluated according to the visible irregularities of the vessel wall. If no collateral flow existed, the mean value of all the other vessel segments in this angiogram was transferred to this occluded vessel segment [19].

The scores were independently assessed by two experienced interventional cardiologists who were blinded to the procedural data and clinical outcomes. The κ for inter-observer variability that was used to estimate the Gensini score and Sullivan Extent score was 0.88, 0.83, respectively, whereas the κ for intra-observer variability was 0.92, 0.90, respectively. In cases of disagreement regarding the Gensini score or Sullivan Extent score, the average of the values from the 2 readers was used as the final value.

### Assessment of covariates, plasma adiponectin, and other biomarkers

Main clinical data including age, sex, body mass index (BMI, were calculated as weight in kilograms divided by the square of height in meters), current smoking (defined as at least 1 cigarette per day for at least 1 year), hypertension, diabetes duration, treatment methods of diabetes were recorded. Fast Blood samples were collected from CAD patients and control subjects, and were stored at −80°C at the day of collection. High-density lipoprotein (HDL) cholesterol, low-density lipoprotein (LDL) cholesterol, triglyceride, glucose, and hemoglobin A1c (HbA1c) were determined. The plasma adiponectin concentration was measured by a commercially available sandwich enzyme-linked immunosorbent assay (Adiponectin ELISA Kit, Otsuka Pharmaceutical Co.Ltd., Tokushima, Japan), the employed assay exhibited inter- and intra-assay CV’s of 3.8–6.5%, depending on the adiponectin concentration of the assayed material. The lower limit of detection was 0.036 μg/ml.

### Determination of adiponectin genotypes

Genomic DNA was extracted from EDTA blood using the E.Z.N.A. ® Blood DNA Mini kit (PEQLAB Biotechnologie Ltd., Erlangen, Germany). Tagging SNPs were selected in the ADIPOQ gene from the HapMap Han Chinese population (HapMap Data Rel 24/phase II, NCBI build 36). There were 11 tagging SNPs (rs266729, rs182052, rs16861205, rs822396, rs12495941, rs7627128, rs1501299, rs3821799, rs3774262, rs1063539, rs12629945), which captured all the 21 SNPs from 1-kb region upstream to 1-kb downstream of the gene [GenBank accession number: NC_000003] with *r*^2^ > 0.8 and minor allele frequency (MAF) > 0.05. Genotypings were carried out by the 5′ nuclease assay using TaqMan® MGB probes on an ABI Prism® 7000 Sequence Detection System (Applied Biosystems, Forster City, CA, USA). TaqMan® MGB probes were allele-specifically labeled with FAM™ and VIC™ reporter dyes, respectively and provided together with corresponding PCR primers by the Assay-on-demand™ service (Applied Biosystems) as a 20 × primer/probe mix. The 5′ nuclease assay was performed in a 15-μL volume, comprising 30–100 ng genomic DNA, 1 × TaqMan® Universal PCR Master Mix (Applied Biosystems), and 1 × primer/probe mix under the following amplification conditions: 2 min at 55°C, 10 min at 95°C and 35 cycles at 90°C for 30 s and 60°C for 1 min. After measurement of the allele-specific fluorescence, SDS software (version 1.1) was used for allelic discrimination. Genotyping quality was tested by including six blinded duplicate samples in each 96-well assay. The average agreement rate of duplicate samples was > 99%.

### Statistical analyses

Continuous variables with normal distribution were expressed as mean ± SD, means were compared by unpaired Student’s t-test. Variables with skewed distributions were ln-transformed before analysis. Categorical variables were presented as percentages and were analyzed by Chi-squared test. Genotype distributions were tested at each polymorphic locus for departure from Hardy-Weinberg equilibrium. Haploview version 4.2 was used to assess linkage disequilibrium (LD). Haplotype frequency and haplotype-tagging SNPs were also determined by means of the algorithms implemented in the Haploview software [20], using 0.05 as the frequency threshold to define common haplotypes. Multivariate logistic or linear regression models were used to estimate the odds ratios (ORs) or regression coefficients under the assumption of an additive effect of allele dosage. Correction for multiple testing was performed by the SNP spectral decomposition method (SNPSpD) [21]. Under this method, the effective number of independent marker loci (M_effLi_) was 7.5, and the experimental-wide significance threshold to keep type 1 error rate at 5% was 0.0086. Correction for testing of multiple phenotypes was not performed when the phenotypes tested were closely related to each other. The P values for interaction were estimated by adjusting for the main effects of all covariates (age, sex, BMI, diabetes duration, current smoking, hypertension, HbA1c, fasting glucose, 2-h post-OGTT glucose, triglyceride, HDL cholesterol, LDL cholesterol) in the multivariate regression models.

Maximum likelihood estimates of haplotype frequencies in CAD patients and control subjects were derived using the expectation-maximization algorithm as implemented in the function HAPLO.EM of the Haplo Stats suite [22]. The association between CAD and common (≥ 0.05) ADIPOQ haplotypes was analyzed using the score statistics proposed by Schaid et al. [22], and implemented in the function HAPLO.SCORE of the Haplo Stats software. This method allows adjustment for covariates (age, sex, BMI, diabetes duration, current smoking, hypertension, HbA1c, triglyceride, HDL cholesterol, LDL cholesterol) and provides a global test of association, as well as haplotype-specific tests. After testing for association with haplotypes, a combination of haplotypes (i.e., a diplotype) was assigned to each individual on the basis of the posterior probabilities of the different phases. The risk of CAD associated with each diplotype relative to CAG/CAG homozygotes was then estimated by multivariate logistic regression analysis as described above. Statistical significances were considered significant at P < 0.05. The SPSS statistical software package ver.16.0 was used for statistical analysis.

## Results

### Subject characteristics and genotyping

Clinical characteristics of the CAD patients and control subjects are listed in Table 1. CAD patients had worse cardiovascular risk profile, longer diabetes duration, worse glycemic control and lower plasma adiponectin level (P < 0.001) than the control subjects. The two groups were similar with respect to LDL cholesterol level and treatments of diabetes.

**Table 1:** Clinical characteristics of control subjects and CAD patients with type 2 diabetes

	**Control Subjects**	**CAD Patients**	**P value**
**n**	**550**	**560**	
**Age (years)**	**61.1 ± 7.9**	**66.0 ± 6.9**	**< 0.001**
**Male subjects (%)**	**42.5**	**55.4**	**< 0.001**
**BMI (kg/m**^**2**^**)**	**22.0 ± 3.4**	**24.3 ± 3.9**	**< 0.001**
**Diabetes duration (years)**	**10.8 ± 3.6**	**12.0 ± 4.4**	**< 0.001**
**Current smoking (%)**	**32.0**	**38.4**	**0.026**
**Hypertension (%)**	**61.8**	**68.9**	**0.013**
**Fasting glucose (mmol/l)**	**6.49 ± 2.71**	**6.86 ± 3.10**	**0.038**
**2-h post-OGTT glucose (mmol/l)**	**7.57 ± 1.21**	**8.40 ± 2.07**	**< 0.001**
**HbA1c (%)**	**7.18 ± 1.01**	**7.53 ± 1.22**	**< 0.001**
**Triglyceride (mmol/l)**	**1.46 ± 0.21**	**1.49 ± 0.26**	**0.023**
**HDL cholesterol (mmol/l)**	**1.33 ± 0.28**	**1.23 ± 0.22**	**< 0.001**
**LDL cholesterol (mmol/l)**	**3.18 ± 0.84**	**3.25 ± 0.83**	**0.135**
**Adiponectin (μg/ml)**	**7.11 (6.90 - 7.33)**	**5.90 (5.69 - 6.12)**	**< 0.001**
**Gensini score**	**13.4 (12.6 - 14.2)**	**22.5 (21.1 - 24.1)**	**< 0.001**
**Sullivan Extent score**	**15.9 (13.9 - 16.6)**	**28.7 (25.5 - 31.1)**	**< 0.001**
**Treatment**			
**Diet only (%)**	**11.3**	**11.4**	**0.935**
**Oral agents (%)**	**57.6**	**53.2**	**0.138**
**Insulin (%)**	**31.1**	**35.4**	**0.131**

Data are expressed as mean ± SD or geometric mean (95% confidence interval). BMI = body mass index; CAD = coronary artery disease; HbA1c = hemoglobin A1C; HDL cholesterol = high-density lipoprotein cholesterol; LDL cholesterol = low-density lipoprotein cholesterol.

Among the 11 SNPs genotyped, the genotype frequencies for SNPs did not differ significantly from those expected under Hardy–Weinberg equilibrium. There were four SNP pairs showing high pairwise LD pattern (rs12495941 and rs7627128, rs3821799 and rs3774262, rs3821799 and rs1063539, rs3774262 and rs1063539; all *r*^2^>0.80).

### Coronary artery disease

Among the 11 SNPs, the minor allele of rs266729 was significantly associated with higher odds of CAD after adjusting for age and sex (P = 0.00095), and correction for multiple testing (Table 2). The association remained significant after further adjusting for BMI, diabetes duration, current smoking, hypertension, fasting glucose, 2-h post-OGTT glucose, HbA1c, triglyceride, HDL cholesterol, LDL cholesterol [OR (95% confidence interval (CI)) = 1.49 (1.10 - 2.16), P = 0.022] (Table 3). In a separate analysis, similar results were obtained under the assumption of dominant or recessive allelic effect (OR (95% CI) = 1.56 (1.18 - 2.09), P = 0.002; OR (95% CI) = 1.90 (1.19 - 3.01), P = 0.005 in full adjustment model, respectively). In forward stepwise multivariate logistic regression, this SNP was a significant independent factor of CAD (Table 3). In this study, the 560 CAD patients and 550 control subjects could provide >80% power (α= 0.05) to detect genetic effects with allelic OR above 1.46 for rs266729.

**Table 2:** Associations of single nucleotide polymorphisms of ADIPOQ with coronary artery disease

**SNPs**	**Alleles major : minor**	**MAF**		**OR (95% CI)**	**P value**
		**Control Subjects**	**CAD Patients**		
**n**					
**rs266729**	**C : G**	**0.240**	**0.338**	**1.64 (1.35 - 2.01)**	0.00095*
**rs182052**	**G : A**	**0.411**	**0.423**	**1.18 (0.98 - 1.52)**	**0.113**
**rs16861205**	**G : A**	**0.158**	**0.147**	**0.82 (0.64 - 1.06)**	**0.126**
**rs822396**	**A : G**	**0.126**	**0.131**	**0.94 (0.72 - 1.23)**	**0.670**
**rs12495941**	**G : T**	**0.372**	**0.398**	**1.04 (0.87 - 1.25)**	**0.572**
**rs7627128**	**C : A**	**0.238**	**0.226**	**0.87 (0.71 - 1.08)**	**0.217**
**rs1501299**	**G : T**	**0.256**	**0.238**	**0.83 (0.67 - 1.03)**	**0.102**
**rs3821799**	**T : C**	**0.392**	**0.421**	**1.07 (0.89 - 1.28)**	**0.482**
**rs3774262**	**G : A**	**0.288**	**0.265**	**0.85 (0.69 - 1.04)**	**0.129**
**rs1063539**	**G : C**	**0.285**	**0.302**	**1.06 (0.87 - 1.30)**	**0.543**
**rs12629945**	**G : A**	**0.198**	**0.183**	**0.83 (0.66 - 1.05)**	**0.116**

MAF, minor allele frequency. All OR and P values are adjusted for age and sex. *P value that can pass multiple testing correction (P < 0.0086).

**Table 3:** OR for the association between CAD and rs266729 SNP in the ADIPOQ

**Parameter**	OR (95% CI) *	**P value**
**AGE**	**1.12 (1.10 - 1.15)**	**< 0.001**
**BMI**	**1.22 (1.17 - 1.27)**	**< 0.001**
**diabetes duration**	**1.06 (1.03 - 1.10)**	**0.001**
**current smoking**	**1.46 (1.08 - 1.97)**	**0.014**
**2-h post-OGTT glucose**	**1.35 (1.23 – 1.47)**	**< 0.001**
**HbA1c**	**1.38 (1.22 - 1.57)**	**< 0.001**
**HDL cholesterol**	**0.32 (0.18 - 0.57)**	**< 0.001**
**rs266729**		
**G†**	**1.49 (1.10 - 2.16)**	**0.022**
**CG‡**	**1.53 (1.08 - 1.92)**	**0.028**
**GG‡**	**2.18 (1.32 - 3.46)**	**< 0.001**

† and ‡ OR are computed using major allele or major allele homozygotes as the reference group, respectively. *Adjusted for age, sex, BMI, diabetes duration, current smoking, hypertension, HbA1c, fasting glucose, 2-h post-OGTT glucose, triglyceride, HDL cholesterol, and LDL cholesterol in stepwise multivariate logistic regression model.

### Plasma adiponectin level

The adiponectin level was negatively correlated with male gender, BMI, hypertension, triglycerides, fasting glucose, 2-h post-OGTT glucose, and HbA1c, and positively correlated with age and HDL cholesterol (all P < 0.05), and was not related to diabetes duration, current smoking, LDL cholesterol (P > 0.05). In forward stepwise multivariate linear regression, male gender, lower age, higher BMI, hypertension, 2-h higher post-OGTT glucose, higher HbA1c, and lower HDL cholesterol were independent factors associated with lower adiponectin level, explaining 37.1% variation in adiponectin level (Table 4). Therefore, in all subsequent analysis of adiponectin level, all models were adjusted for age, sex, BMI, hypertension, 2-h post-OGTT glucose, HbA1c, and HDL cholesterol.

**Table 4:** Stepwise multivariate linear regression for adiponectin level (ln-transformed)

**Parameters**	β*	**P value**
**age**	**0.008**	**< 0.001**
**male gender**	**- 0.181**	**< 0.001**
**BMI**	**- 0.041**	**< 0.001**
**hypertension**	**- 0.136**	**< 0.001**
**2-h post-OGTT glucose**	**-0.011**	**0.044**
**HbA1c**	**- 0.025**	**0.003**
**HDL cholesterol**	**0.191**	**< 0.001**
**R**^**2**^	**0.371**	

* Adjusted for age, sex, BMI, diabetes duration, current smoking, hypertension, HbA1c, fasting glucose, 2-h post-OGTT glucose, triglyceride, HDL cholesterol, and LDL cholesterol in the stepwise model.

**Figure 1:**
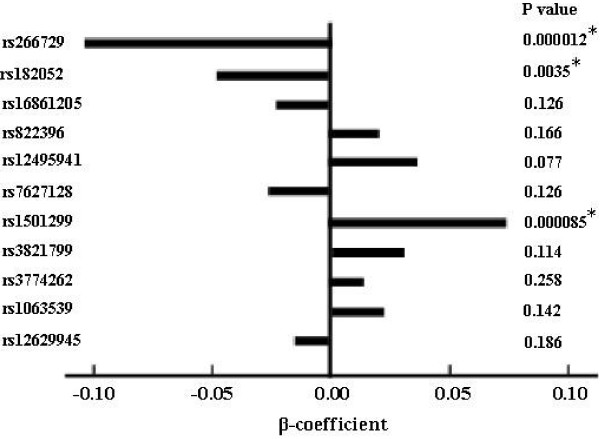
**Associations of SNPs with adiponectin level.** The bar shows the magnitude of β-coefficients in multivariate linear regression analysis of adiponectin level (ln-transformed) after adjusting for age, sex, BMI, hypertension, 2-h post-OGTT glucose, HbA1c, and HDL cholesterol. * P values that can pass multiple testing correction (P < 0.0086).

Several genetic variants were significantly associated with adiponectin level after adjusting for the above covariates (Figure 1). The minor alleles of rs266729 and rs182052 were significantly associated with lower level (β = −0.101, P < 0.0001 and β = −0.044, P = 0.0035 respectively), whereas the minor alleles of rs1501299 were significantly associated with higher level (β = 0.073, P < 0.0001).

### Haplotype analysis

Haplotypes were constructed using the rs266729, rs182052, and rs1501299 as these SNPs showed significant association with adiponectin level. There were five common haplotypes with frequency >5%, namely, CGG (32.8%), CGT (15.6%), CAG (19.4%), GAG (17.9%), and GGG (5.2%).

**Figure 2:**
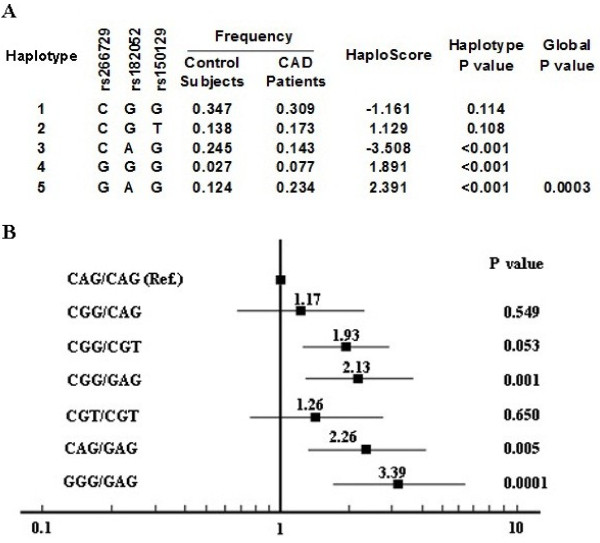
**Haplotypes in the ADIPOQ locus and cardiovascular risk. A**: Haplotype frequencies in Control subjects and CAD patients. Positive and negative scores denote an association with increased and decreased risk of CAD, respectively. Haplotype-specific P values are reported along with the global P value. **B**: OR of CAD associated with different haplotype combinations (diplotypes). OR are indicated by the squares and 95% CI by the lines.

Consistent with the individual SNP analyses, a significant association with CAD was observed for the haplotypes. Figure 2A shows the maximum likelihood estimates of the haplotypes in the combined case and control subjects along with their score statistics for association with CAD as computed by the HAPLO.SCORE program [22]. Haplotype distributions were significantly different in case and control subjects (global P = 0.0003). Haplotype CAG, which exactly corresponds to the major allele of rs266729, was associated with protection from CAD (P < 0.001), whereas haplotype GAG, exactly corresponding to the minor allele of rs266729, was associated with predisposition (P < 0.001). Despite the low frequency of GGG haplotype, a predisposing effect was also observed for this haplotype, and the P value did reach significance (P < 0.001). When diplotypes were assigned to each subject, there were seven common diplotypes with frequency >5%, haplotypes GGG and GAG combination were significantly associated with an increased risk of CAD (Figure 2B). The ORs of CAD for these genotypes compared with CAG/CAG homozygotes ranged from 2.13 to 3.39. The ORs were lower and did not differ significantly from 1.0 when haplotypes CGT and CGT, CGG and CAG combinated as diplotypes (Figure 2B). We also noticed that CGG/CGT diplotype had an increased risk for CAD, although the P value was marginally significant (P = 0.053).

### Angiographical severity and extent of coronary atherosclerosis

**Figure 3:**
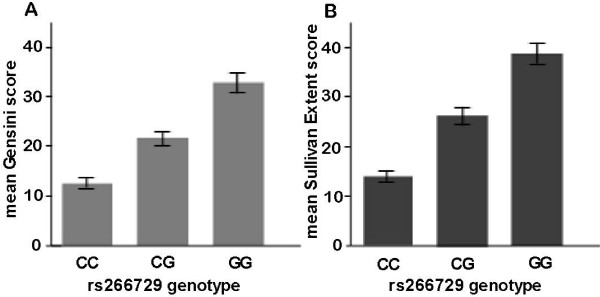
**Coronary artery disease severity by rs266729 genotype.** CAD severity is illustrated here as (**A**) the unadjusted geometric mean of Gensini score (P < 0.001) and (**B**) the unadjusted geometric mean of Sullivan Extent score (P < 0.001). The error bar shows the 95% CI of the geometric mean.

The association of genetic variants in the ADIPOQ gene with the angiographical severity and extent in CAD patients and control subjects was evaluated by investigating Gensini and Sullivan Extent scores according to adiponectin genotypes. Gensini score (ln-transformed) increased significantly from the rs266729 genetype CC over the CG to the GG (Data are expressed as geometric mean (95% CI), 12.4 (11.6 - 13.2) vs. 22.2 (21.0 - 23.5) and 34.1 (31.5 - 36.9), P < 0.001, respectively), and Sullivan Extent score (ln-transformed) increased significantly from the rs266729 genetype CC over the CG to the GG (Data are expressed as geometric mean (95% CI), 14.6 (12.5 - 15.1) vs. 25.4 (22.5 - 27.3) and 38.6 (33.8 - 41.7), P < 0.001, respectively) (Figure 3). Moreover, the minor G allele was significantly associated with higher Gensini score (β = 0.139, P < 0.001) and Sullivan Extent score (β = 0.107, P < 0.001) after adjusting for age, sex, BMI, diabetes duration, current smoking, hypertension, 2-h post-OGTT glucose, HbA1c, triglyceride, HDL cholesterol, and LDL cholesterol in stepwise multivariate linear regression model. The association remained significant under the assumption of dominant or recessive allelic effect even after adjusting for the above covariates (Gensini score: β = 0.152, P < 0.001, β = 0.163, P < 0.001, respectively; Sullivan Extent score: β = 0.131, P < 0.001, β = 0.142, P < 0.001, respectively).

## Discussion

In this study, we have demonstrated that three genetic variants, namely rs266729 (−11377C>G), rs182052 (−10066G>A), and rs1501299 (+276G>T) are associated with adiponectin level, with the effect being stronger for rs266729 and rs1501299. The present study also indicates the correlation between the variants of rs266729 and angiographically-diagnosed CAD in type 2 diabetes in Chinese population, and this is the first investigation demonstrating that the minor allele of rs266729 is associated with the angiographical severity and extent of coronary atherosclerosis. This effect appears to be due to allelic differences in ADIPOQ, which may influence the antiatherogenic effects of adiponectin on target tissues. These results confirm and extend the evidence implicating adiponectin as a physiological modulator of atherogenesis and point to variability in the ADIPOQ gene as a key regulator of this effect.

Among these SNPs, rs266729 is located in the gene promoter region, whereas rs182052 and rs1501299 are located in the intron 1 and intron 2 region. When compared with other previous studies, there seems to be ethnic difference in the associations of genetic variants with adiponectin level. In the Genetic Epidemiology of Metabolic Syndrome Study, the SNPs most strongly associated with adiponectin were rs3774261 and rs6773957 in ADIPOQ gene in Northern and Western European populations [14]. In the Framingham Offspring Study [15], the SNPs of rs17300539, rs822387 (which both are located in promoter region), and rs6773957 (which is located in 3'-untranslated region) were associated with adiponectin levels, which were not found in our subjects.

According to the Alibaba 2.1 program [23], a loss of a Sp1-binding site and gain of a CCAAT/enhancer-binding protein (C/EBP) β-binding site are resulted from the presence of the minor allele of the SNP rs182052. These two binding sites are involved in adipocyte differentiation [24]. Previous study has demonstrated that the first intron of the human ADIPOQ gene contains a gene expression enhancer element, which responds to C/EBP α, but not to C/EBP β [25]. Therefore, to investigate whether this enhancer element could also respond to Sp1 is of great interests.

While the effect of ADIPOQ variability on CAD risk appears to be mediated by differences in gene expression, the identities of the sequence variants that are responsible for this effect remain to be further investigated. The observation that the five haplotypes fall into more than two risk classes suggests the involvement of multiple polymorphisms, either the tagging SNPs or variants in linkage disequilibrium with them, interacting with each other. It has been reported that the minor G allele of SNP rs266729 alters DNA-binding activity, leading to lower basal and inducible promoter activity in mouse 3 T3-L1 adipocytes [26]. Other findings also reveal that this SNP destroys the binding site of transcriptional stimulatory protein, Sp1 [27]. This might be a reasonable explanation for the association between rs266729 SNP and CAD.

ADIPOQ polymorphisms may affect cardiovascular risk through various mechanisms involved in adiponectin. Adiponectin suppresses endothelial adhesion molecule expression, vascular smooth muscle cell proliferation, and macrophage-to-foam cell transformation as well as tumor necrosis factor-alpha and interleukin-6 production by macrophages in vitro [28,29]. It has been reported that the adiponectin-knockout mice exhibits enhanced neointimal thickening after vascular injury [30]. Administration of recombinant adenovirus expressing human adiponectin to apoE-deficient animals causes a 30% reduction in the formation of atherosclerotic lesions in the absence of any effects on metabolic traits [31]. These findings suggest that adiponectin has a wide range of antiatherogenic property and acts as an endogenous mediator of vascular and metabolic diseases. In our study, CAD patients had lower plasma adiponectin level than the control subjects, which is aligned with the result from a recent meta-analysis [32].

Although rs266729 has been reported to be associated with diabetes, severe forms of obesity, carotid intima media thickness, and other cardiovascular risks [3,4,33-37], the role of adiponectin gene rs266729 (−11377C>G) polymorphisms in angiographic CAD was seldom explored. In our investigation, the presence of the G allele of the rs266729 is strongly associated with an increased prevalence of coronary heart disease. This is aligned with the findings by Hoeffel et al. [16]. A recent meta-analysis also showed that the associations between rs266729 in the ADIPOQ and cardiovascular disease were significant [38]. But there are some differences from Zhong’s study [39], it was reported that rs266729 of the adiponectin gene was not associated with CAD (OR =1.24, 95%CI: 0.91-1.69, p= 0.20) in a 435 Chinese population study, but female carriers of allele G at rs266729 had a higher risk of CAD compared with allele C carriers (OR = 1.30, 95% CI: 1.09-2.64, p = 0.02). Moreover, it isn’t also consistent with Chen’s study [40], which revealed that genetic polynmrphism in rs266729 was not associated with coronary artery disease in a 517 Chinese subjects. However, we noticed that the major limitation of the studies was the number of the patients. Several studies arising from the HapMapII data such as the Wellcome trust case control consortium reported required sample sizes of several thousands even if the MAF is 0.5 and the assumed OR larger than 1.5 and adjusted p of 10E-8 [41,42]. On the other hand, the association between the −11377 C>G proximal promoter polymorphism and CAD hasn’t also been detected in patients with type 2 diabetes in Caucasian population [43]. There also seems to be ethnic difference in the associations of genetic variants with CAD. Interestingly, our data also reveal that the minor G allele of SNP rs266729 is associated with the severity and extent of coronary atherosclerosis when the angiographic extent was evaluated by Gensini score and Sullivan Extent score in Chinese type 2 diabetes. We found that the heterozygous CG genotype and the homozygous GG genotype had higher Gensini score and Sullivan Extent score, and this difference remained significantly even after controlling for covariates. Although the exact biological mechanism of this association remains to be explored, our study provides credible evidence that the rs266729 polymorphism may contribute to the etiology of the severity of coronary atherosclerosis and plays an important role in the atherosclerotic process.

The adiponectin rs1501299 G>T variant was positively related with an increased risk of CAD, and the CAD patients had lower adiponectin levels which were not affected by the different genotypes of rs1501299 in Gui’s study [44]. However, there was an association between rs1501299 G>T and adiponectin levels in our study. But we should notice that only 35% of subjects in Gui’s study have detected the adiponectin levels, it could reside in the sample size not large enough to investigate this precise issue. The exact reasons were not clear, the difference of the study population may in part explain the difference of the results. Enlarging the sample size and performing a prospective study would make the conclusions more convincing.

### Study limitations

Some limitations of our study should be acknowledged. As a potential limitation, population stratification may influence the observed associations. However, our population is racially homogeneous, with the whole of the participants being Han. On the other hand, our study population was highly selected, and therefore our results may not be necessarily applicable to other populations. Another limitation is that the two groups had some differences in the baseline characteristics, which may affect the results. We have attempted to minimize the effect of these differences by multivariate analysis. Moreover, it must be emphasized that this was a cross-sectional study, which might be susceptible to bias due to possible effects of genetic variants on survival. Thus, our findings must be confirmed in prospective studies before ADIPOQ polymorphisms can be considered as predictors of CAD in type 2 diabetes. Finally, Receptors for adiponectin have been characterized that mediate effects of adiponectin in various tissues and may have a higher risk of cardiovascular disease [45]. Advanced investigation should be conducted to demonstrate whether the receptors for adiponectin, such as ADIPOR1 and ADIPOR2, have an impact on the risk of developing type 2 diabetes and CAD in a special race.

## Conclusions

In conclusion, several genetic variants in the ADIPOQ gene are associated with adiponectin level in this Chinese population. Among these variants, a C to G allele substitution of the rs266729 SNP is associated with a significantly higher risk of CAD in type 2 diabetes, and this SNP is also associated with the angiographical severity of coronary atherosclerosis. Our study has provided further evidence for a role of adiponectin in the development of CAD.

## Abbreviations

ADIPOQ: Adiponectin gene; CAD: Coronary artery disease; BMI: Body Mass Index; SNPs: Single nucleotide polymorphisms; HDL cholesterol: High-density lipoprotein cholesterol; LDL cholesterol: Low-density lipoprotein cholesterol; HbA1c: Hemoglobin A1c; LD: Linkage disequilibrium; MAF: Minor allele frequency.

## Competing interests

The authors declare that they have no competing interests.

## Authors’ contributions

All authors listed on the manuscript participated in the design and coordination of the study and made substantial contribution to the intellectual content of the project to be included as authors. All authors read and approved the final manuscript.
